# Association of Eating Disorders with Recurrent Pain in Adolescent Girls

**DOI:** 10.1192/j.eurpsy.2022.971

**Published:** 2022-09-01

**Authors:** N. Semenova, H. Slobodskaya, E. Rezun

**Affiliations:** 1 Scientific Research Institute of Medical Problems of the North, Department Of Child’s Physical And Mental Health, Krasnoyarsk, Russian Federation; 2 Federal State Budgetary Scientific Institution «Scientific Research Institute of Neurosciences and Medicine», Department Of Child Development And Individual Differences, Novosibirsk, Russian Federation

**Keywords:** Adolescents, recurrent pain, Eating Disorders, girls

## Abstract

**Introduction:**

Eating disorders (ED) are associated with other mental illnesses, but the association of ED with pain is less well understood.

**Objectives:**

To study the association of ED with headache and abdominal pain in adolescent girls.

**Methods:**

In 2015-2018, 917 girls aged 12-17 were examined using the Body Image and Eating Distress scale (Koskelainen et al., 2001) and questions about frequency of recurrent headache and abdominal pain over the past six months. Adolescents were divided into three groups: girls with eating disorders (ED, n = 20); subthreshold eating disorders (SED, n = 88); and a control group (CG, n = 809).

**Results:**

Headaches of varying frequency are were reported by 80% of girls with ED, 70.4% of girls with SED and 52.2% of CG girls. Frequent headaches (every week) were reported by 60% of girls with ED, 40.9% of girls with SED, and 29.9% of CG girls (χ2 = 20.21, p = 0.003). Recurrent abdominal pain was reported by 65% of girls with ED, 56.8% of girls with SED, and by 46.6% of CG girls. Weekly abdominal pain affected 30% of girls with ED, 20.4% of girls with SED and 12.9% of CG girls. Combined weekly pain were commoner in girls with eating distress (in 20% of girls with ED and in 12.5% with SED) than in CG (7.7%, χ2 = 3.92, p = 0.04).

**Conclusions:**

Eating disorders in adolescents are often associated with pain, which can lead to late recognition of the disease, worsening its course and prognosis.

**Disclosure:**

The study was supported by the Russian Science Foundation grant # 21-15-00033

